# Imaging Circulating Tumor Cells in Freely Moving Awake Small Animals Using a Miniaturized Intravital Microscope

**DOI:** 10.1371/journal.pone.0086759

**Published:** 2014-01-31

**Authors:** Laura Sarah Sasportas, Sanjiv Sam Gambhir

**Affiliations:** 1 Department of Radiology, Molecular Imaging Program, Stanford University, Stanford, California, United States of America; 2 Department of Bioengineering, Stanford University, Stanford, California, United States of America; Wayne State University, United States of America

## Abstract

Metastasis, the cause for 90% of cancer mortality, is a complex and poorly understood process involving the invasion of circulating tumor cells (CTCs) into blood vessels. These cells have potential prognostic value as biomarkers for early metastatic risk. But their rarity and the lack of specificity and sensitivity in measuring them render their interrogation by current techniques very challenging. How and when these cells are circulating in the blood, on their way to potentially give rise to metastasis, is a question that remains largely unanswered. In order to provide an insight into this "black box" using non-invasive imaging, we developed a novel miniature intravital microscopy (mIVM) strategy capable of real-time long-term monitoring of CTCs in awake small animals. We established an experimental 4T1-GL mouse model of metastatic breast cancer, in which tumor cells express both fluorescent and bioluminescent reporter genes to enable both single cell and whole body tumor imaging. Using mIVM, we monitored blood vessels of different diameters in awake mice in an experimental model of metastasis. Using an in-house software algorithm we developed, we demonstrated *in vivo* CTC enumeration and computation of CTC trajectory and speed. These data represent the first reported use we know of for a miniature mountable intravital microscopy setup for *in vivo* imaging of CTCs in awake animals.

## Introduction

Metastasis, the cause for 90% of cancer mortality, [1] is a complex and poorly understood process, [2] involving the invasion of blood vessels by circulating tumor cells (CTCs). For metastatic breast cancer, many recent studies have highlighted the prognostic value of CTCs [3], [4] and their clinical potential as predictive biomarkers for response to therapy. [5], [6] However, heterogeneous results have been obtained when comparing CTCs enumerated in the same patient blood samples using different CTC detection technologies. [7]–[11]

Biopsy of the primary tumor can be a painful procedure for the patient and might be hard to obtain depending on the location of the primary tumor. Primary tumor biopsies are routinely used in the clinics to stratify patients and inform therapy decisions. However, this decision is complicated by the heterogeneity in the primary tumor as well as a genetic disparities between metastases and primary tumor. [12] As opposed to cells from the primary tumor mass, CTCs can potentially originate from the primary tumor or from the metastases and can potentially contribute to metastases or return to the primary tumor (a process known as “self-seeding”). [13] Therefore CTCs might be more representative of the disease as a whole as compared to primary tumor biopsies and seem very promising as a painless “liquid biopsy” of the tumor. [14]

However, very little is known about how CTCs reflect the state of the primary tumor or how much they can reveal about the metastatic potential of a patient’s tumor. For decades, invasion was believed to be a relatively later step in tumor progression [15] but recent studies have shown that this process may happen at a relatively early stage, even before the main tumor has been detected by current imaging techniques. [16], [17] Understanding the appearance and dynamics of CTCs during the course of tumor development may help to supplement existing biomarker and imaging-based strategies to improve management of metastatic breast and other cancers.

In the past decade, a variety of techniques have been developed to interrogate CTCs, both *in vitro* in patient blood samples [18]–[22] and *in vivo* by imaging mouse blood vessels using conventional benchtop intravital microscopy or custom-made “*in vivo* flow cytometers”. [23], [24] However, none of these techniques have been able to track the continuous dynamics of CTCs for the following two reasons: (1) Many techniques relying on epithelial markers (e.g. EpCAM) to detect or capture CTCs may miss the most invasive CTCs which have shed those markers when undergoing an epithelial-to-mesenchymal transition (EMT), [25], [26] (2) More importantly, as CTCs are very rare events – as low as 1 CTC per billion of blood cells [27] – their dynamics are likely to be stochastic over time. We hypothesized that there could be peaks of CTCs shedding corresponding to specific events in tumor development, such as the angiogenic switch. [28] However, current *in vitro* CTC detection techniques are limited by blood sample volume and sampling frequency. In the clinical setting, 7.5 mL of patient blood (0.15% of the total blood volume) is typically sampled at baseline (before therapy), then after each full course of therapy. In the preclinical setting, veterinary guidelines usually limit blood sampling to a weekly 100 µL sample in mice (5% of the total blood volume). *In vivo* techniques are limited by the amount and duration of anesthesia that a tumor-bearing animal can physiologically support. Veterinary guidelines recommend that the animals be anesthetized less than 2h, at a maximum frequency of 2–3 times a week, for a duration of maximum of 2 weeks. [29] Therefore, current techniques are not capable of fully evaluating the complex long-term dynamics of CTCs during tumor progression. These dynamics can only be deconvoluted by assessing CTCs *in vivo* continuously over many days, to capture the full spectrum of rare events over the time-course of tumor development. For this purpose, a new method is required that circumvents the need for anesthesia requirement, and allows continuous monitoring of blood vessels *in vivo*.

Intravital microscopy (IVM) is a molecular imaging technique that allows microscopic imaging of cellular and molecular processes in living subjects with exquisite temporal and spatial resolution. [30], [31] In the past two decades, this technique has enabled key biological insights in the fields of immunology, neurobiology, and tumor biology. [32] Recently, a benchtop intravital microscopy setup termed “in vivo flow cytometer” was developed to interrogate circulating tumor cells in anesthetized animal models. [23], [24] Our collaborators, Ghosh et al. recently demonstrated the feasibility of miniaturization of a conventional epifluorescence microscope setup and its application to *in vivo* imaging of awake animals. [33]

In this study, we developed an experimental imageable mouse model of metastatic breast cancer and implemented a novel miniature mountable intravital microscopy method that allows real-time continuous monitoring of CTCs as they circulate in superficial skin blood vessels of an awake mouse. Using this method, we monitored blood vessels of different diameters in awake mice in an experimental model of metastasis. Using an in-house software algorithm, we demonstrated *in vivo* CTC enumeration and computation of CTC trajectory and speed. These data represent the first reported use we know of for a miniature mountable intravital microscopy setup for *in vivo* imaging of CTCs in awake animals.

## Materials and Methods

### Cell culture

The mouse 4T1 cell line (purchased from ATCC, Catalog #CRL-2539) and mouse 4T1-GL cell line (4T1 cell line expressing the GFP-Luc2 construct) are metastatic mouse breast cancer cells and were cultured in Dulbecco’s modified Eagle medium High-Glucose (DMEM, Invitrogen) supplemented with 10% heat-inactivated fetal bovine serum (FBS), 100 units/mL penicillin G-sodium and 100 µg/mL streptomycin sulfate. They were grown to 90% confluency, then rinsed once with phosphate buffered saline (PBS) followed by cell dissociation using 0.05% trypsin-EDTA at 37°C for 5min.

**Green fluorescent dye labeling of breast cancer cells**

4T1 cells were harvested by trypsinization, then washed by centrifugation and re-suspended in a solution of prewarmed PBS containing 10 µM of Vybrant CFDA SE Cell Tracker (Invitrogen Vybrant CFDA SE Cell Tracer Kit, V12883). After incubation at 37°C for 15 minutes, the cells were pelleted by centrifugation and incubated in pre-warmed medium at 37°C for another 30 minutes before being finally washed and re-suspended in 100-200 µL PBS for systemic injection.

### Lentiviral Reporter Gene Construct

Luciferase 2 (Luc2)-eGFP (LG), linked by “gcctctgctgcctctgcc” which encodes 6 amino acids (VSAVSA), was kindly provided by Dr. Ramasamy Paulmurugan (Stanford University). This vector contains the Ubiquitin C promoter sequence.

### Establishment of a highly expressing stable cell line

Upon transfection of the 4T1 cell line using a lentiviral construct containing a bifusion reporter of enhanced green fluorescent protein (eGFP) and firefly luciferase-2, the transfected cells were harvested and selected by two round of Fluorescence Activated Cell Sorting (BD Biosciences FACSAria II cell sorter): based on GFP fluorescence, the brightest 5% cells were selected from the mixed population. The cells were passaged in cell culture and levels of expression of the construct at several passages were checked by FACS analysis.

### Murine experimental metastasis model

Female Nu/nu mice were purchased from Charles River Laboratories (Wilmington, Massachusetts). All animal studies were approved by the Stanford University Institutional Animal Care and Use Committee. 1×10^6^ 4T1-GL metastatic breast cancer cells were freshly harvested and re-suspended in a 100 µL PBS solution then injected intravenously via the tail vein over 20 seconds using a 28 gauge syringe.

### Blood collection

Blood samples were collected from the animals by submandibular bleeding. For 12 days, every 3 days, a sample of 100 µL was collected into K2-EDTA-coated tubes via a blood collection funnel (Greiner Bio-One Minicollect). Following blood collection, 100 µL PBS was injected subcutaneously in each animal.

### Window Chamber Implantation

Animals were deeply anesthetized by intraperitorneal injection of a 300 µL mixture of 1 mg/mL of Xylazine and 10 mg/mL Ketamine. Dorsal hair was removed using hair clippers and depilatory cream. Following this, medium-sized titanium dorsal skinfold window chambers (APJ Trading, Cat# MD100) were surgically implanted on the back of the animals following a previously described surgery procedure. [34] Briefly, following the midline, a titanium frame was sutured to the right side of the dorsal skin using surgical sutures (Blue Polypropylene, 5-0, FS-2) (Med Rep Express, MA). Both layers of the skin flap were punctured in two instances for two stainless steel screws. A window was made into the left side of the skin by removing a round-shaped epidermal layer, which was replaced by a sterile 12 mm-diameter glass coverslip secured by an O-ring. Following this, both frames were screwed together, and sutured to the skin flap. The animals were allowed to recover over a period of 3–4 days.

### Bioluminescence Imaging

For bioluminescence imaging, 100 µl of 30 mg/ml D-Luciferin (Biosynth AG, Switzerland) was injected intraperitoneally before placing mice under 1–2% inhaled isofluorane anesthesia. Bioluminescence signal was monitored using the IVIS system 200 series (Xenogen, Alameda, CA, USA), consisting of a highly sensitive, cooled CCD camera. Living Image software (Xenogen, Alameda, CA, USA) was used to draw regions-of-interest (ROI) and integrate the total bioluminescence signal in each ROI. Data were analyzed using average photon flux emission (photons/second/cm^2^/sr) in the ROIs and normalized to background signal. Organs were harvested and immediately soaked in a 3 mg/mL solution of D-Luciferin for 5 minutes prior to BLI imaging.

### Image processing MATLAB algorithm for vessel edge definition, CTC detection by shape/size and CTC counting

•Read movie

•Green Channel selection

•Background subtraction

•Appropriate thresholding

•Define cell-like objects (based on edges, shape and size)

•Label and count objects

•Compute trajectory

•Overlay vessel and cell edges

### Statistical analysis

Results were expressed as mean ± standard error of the mean, unless indicated otherwise. An unpaired, 2-tailed Student’s t test was used to calculate P values. P values ≤0.05 were considered statistically significant and reported as asterisks: * for P ≤ 0.05, ** for P ≤ 0.01, *** for P ≤ 0.001 and **** for P ≤ 0.0001.

### Ethical statement

This study was performed in strict accordance with the recommendations in the Stanford's Administrative Panel for Laboratory Animal Care (APLAC) and this study was specifically approved by Stanford University's APLAC board (APLAC #21127, APLAC #11581). All surgeries were performed under anesthesia and all efforts were made to minimize suffering.

## Results

### Development of a dual-modality imageable mouse model of breast cancer metastasis

**Figure 1:**
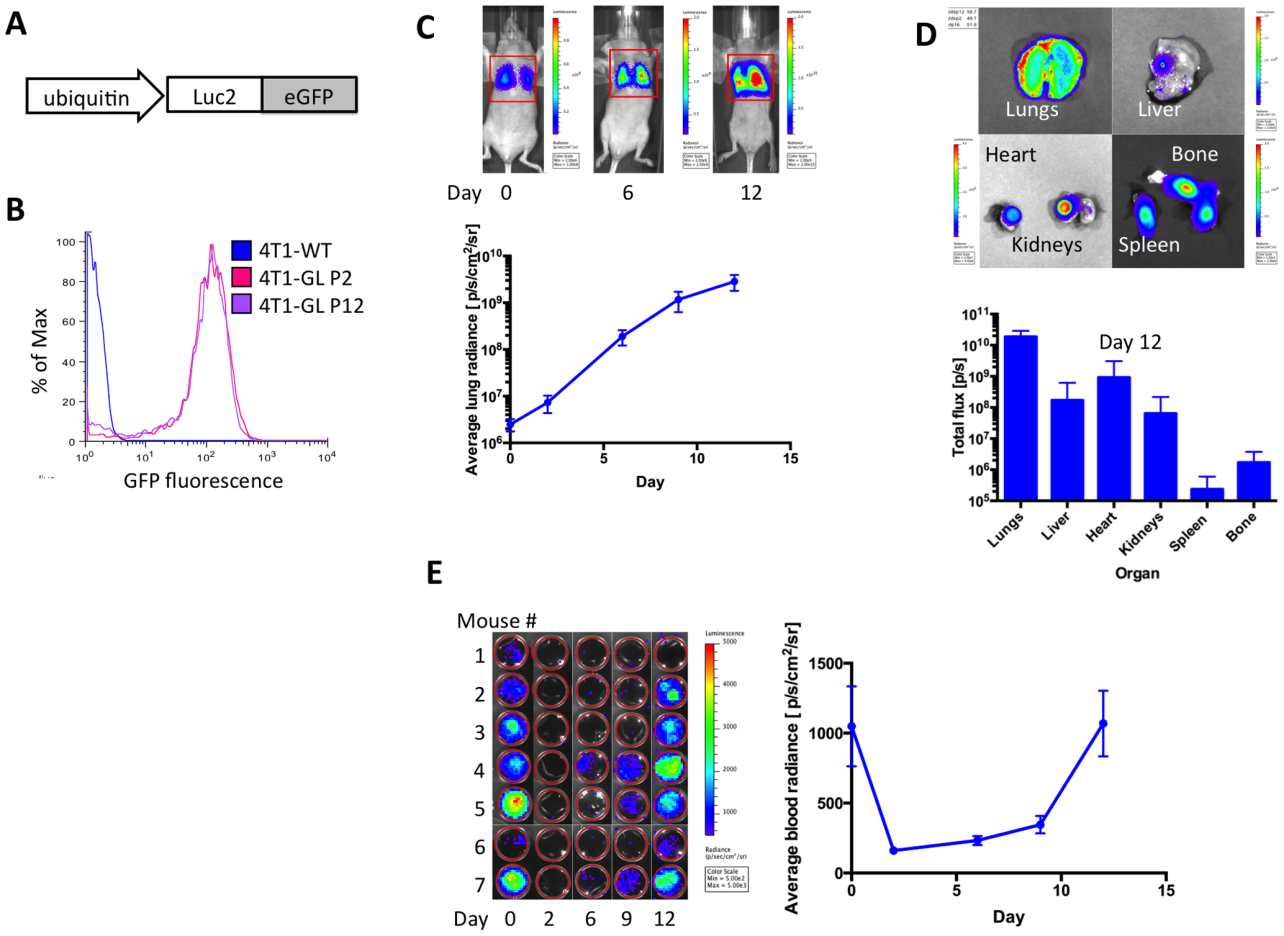
Experimental mouse metastatic breast cancer model. (**A**) Schematic of lentiviral construct comprising a fusion reporter gene (Luciferase-2 and enhanced GFP) under the control of the ubiquitin promoter, used to establish the imageable metastatic mammary carcinoma cell line 4T1-GL. (**B**) FACs analysis of GFP fluorescence, comparing the stable cell line 4T1-GL at passage 2 and passage 12 (resp. P2 and P12) to wild-type 4T1 cells (4T1-WT). (**C**) Metastatic tumor growth in the lungs as monitored non-invasively by Bioluminescence (BLI) imaging, following a systemic injection of 1×10^6^ 4T1-GL cells via the tail vein (n = 7). (**D**) Biodistribution of metastatic cells, 12 days after systemic injection (n = 7) in the following organs: Lungs, Liver, Heart, Kidneys Spleen, Bone marrow, and corresponding quantification of BLI signal per organ (n = 7). (**E**) CTCs in 100 µL blood samples of mice (n = 7) at various times from day 0 (immediately after injection) to 12 days after injection and corresponding signal quantification. Positive BLI signals correspond to <20 CTCs/100 uL of blood.

We transfected the murine metastatic carcinoma cell line 4T1 using a lentiviral construct containing a bifusion reporter of enhanced green fluorescent protein (eGFP) and firefly luciferase-2 (Luc2, Fig. 1A). The fusion protein gene is placed under the control of the ubiquitin promoter harboring longer and sustained expression of the transgene for long-term cellular imaging. [35] Using two rounds of fluorescence activated cell sorting (FACS), we established a stable cell line (denoted 4T1-GL), in which 77.1% of the cells express high levels of the bifusion reporter gene, as demonstrated by GFP fluorescence (Fig. 1B). This high level of fluorescence is retained throughout 10 passages as demonstrated by FACS analysis of the GFP fluorescence of the 4T1-GL cell line at passage 2 (P2) and 12 (P12, Fig. 1B). The cells labeled with the reporter behaved similarly to the parental wild-type cell line in terms of growth rate and harbored the same microscopical morphology (data not shown).

### Distribution of systemically injected CTCs in the 4T1-GL metastatic breast cancer model

Following intravenous injection of 1×10^6^ 4T1-GL via the tail vein, we were able to monitor metastatic burden in the lungs of mice (n = 7) by BLI, which exponentially increased over 12 days (Fig. 1C). We also measured BLI signal in 100 µL blood samples obtained by submandibular bleeding (Fig. 1E). We observe high numbers of 4T1-GL cells circulating in the blood at the time of tail-vein injection, that disappear in the following days after injection. Interestingly we observed a peak of re-circulation of CTCs at day 9–11, as well as day 15, where we performed a terminal bleeding (500 µL) in all animals. Multiple metastases in multiple organs (lungs, liver, heart) were observed by *ex vivo* BLI at the end of the study on day 15 (Fig. 1D). These results demonstrate that systemic injection of CTCs lead to a strong lung metastatic burden and that recirculation of CTCs is leading to secondary sites of metastasis over an 11-day period. From this thorough study evaluating CTCs and the subsequent metastatic burden in a mouse model, we concluded that our experimental 4T1-GL mouse metastatic model is amenable for investigating CTC circulation *in vivo*, using a novel mountable miniature intravital microscopy system described next.

### Development of a mountable intravital microscopy (mIVM) system

**Figure 2:**
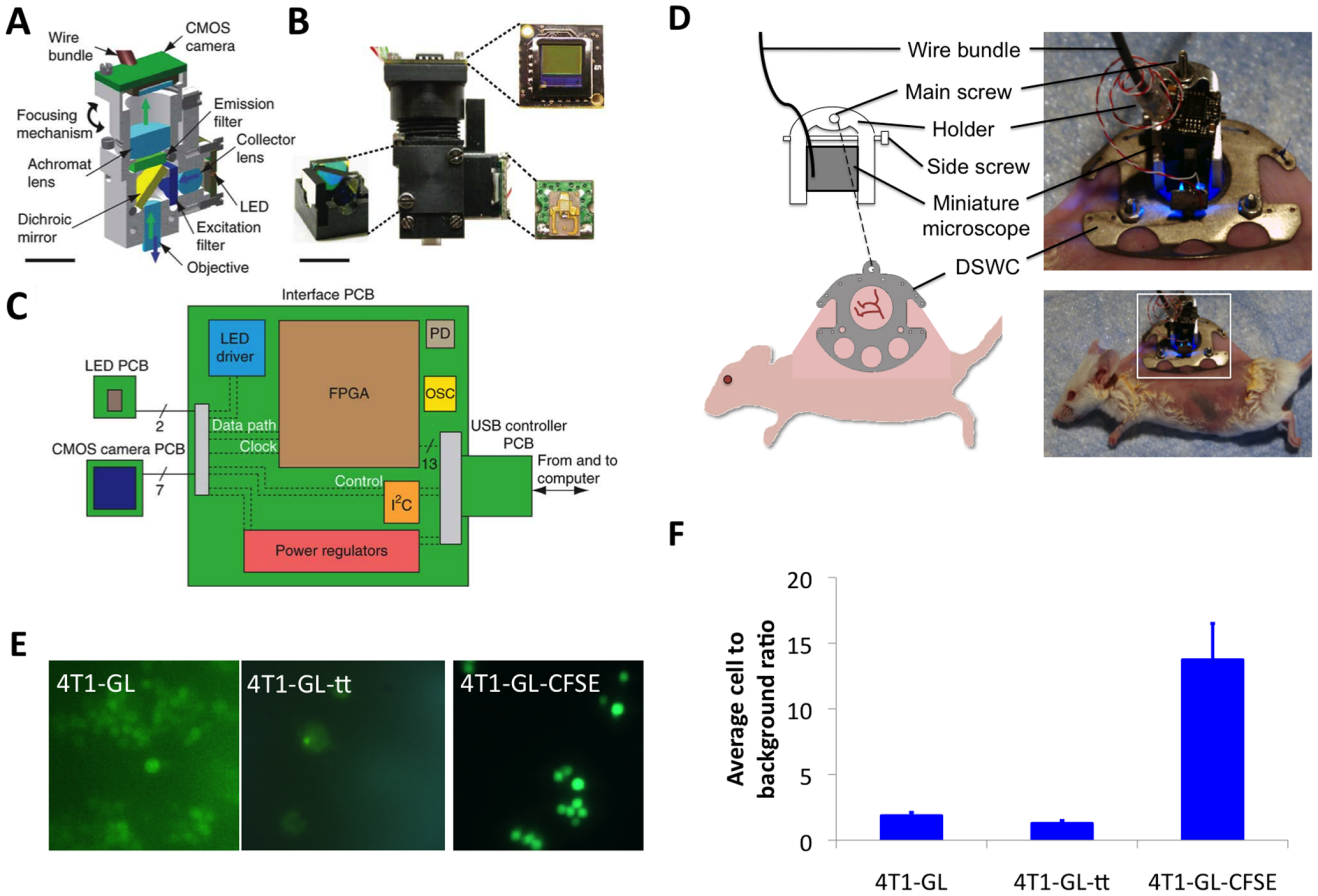
Miniature mountable intravital microscopy system design for *in vivo* CTCs imaging in awake animals. (**A**) Computer-assisted design of an integrated microscope, shown in cross-section. Blue and green arrows mark illumination and emission pathways, respectively. (**B**) Image of an assembled integrated microscope. Insets, filter cube holding dichroic mirror and excitation and emission filters (bottom left), PCB holding the CMOS camera chip (top right) and PCB holding the LED illumination source (bottom right). The wire bundles for LED and CMOS boards are visible. Scale bars, 5 mm (**A**,**B**). (**C**) Schematic of electronics for real-time image acquisition and control. The LED and CMOS sensor each have their own PCB. These boards are connected to a custom, external PCB via nine fine wires (two to the LED and seven to the camera) encased in a single polyvinyl chloride sheath. The external PCB interfaces with a computer via a USB (universal serial bus) adaptor board. PD, flash programming device; OSC, quartz crystal oscillator; I^2^C, two-wire interintegrated circuit serial communication interface; and FPGA, field-programmable gate array. (**D**) Schematic of the miniature mountable intravital microscopy system and corresponding images. The miniature microscope is attached to a dorsal skinfold window chamber via a lightweight holder. (**E**) mIVM imaging of cells in suspension in a glass-bottom 96-well plate. 4T1-GL cells; 4T1-GL cells that have been transiently transfected with the Luc2-eGFP DNA to enhance their fluorescence (4T1-GL-tt); 4T1-GL cells that have been labeled with the bright green fluorescent CFSE dye (4T1-GL-CFSE). (**F**) Quantification of the cell to background green fluorescence for the three cell types described in (E) for n = 3 field of view, average ±standard deviation. **Fig. 2** (**A**)**,** (**B**)**,** (**C**) reprinted by permission from Macmillan Publishers Ltd: Nature Methods (Ghosh, K. K. *et al.* Miniaturized integration of a fluorescence microscope. *Nat Meth* **8,** 871–878 (2011)), copyright 2011.

Ghosh et al. have recently introduced a miniature integrated fluorescence microscope, made from mass-producible elements and capable of *in vivo* high speed (100 Hz) cellular imaging and imaging of capillaries microcirculation. [33] This miniature intravital microscope incorporates a conventional epifluorescence microscope architecture into a ∼2.4 cm^3^ housing, without any fiber bundle coupling, allowing for imaging of freely moving awake animals. The excitation light source is a blue LED, with excitation light collected on a drum lens, filtered by a 480/40 nm bandpass filter, reflected off a dichroic mirror and delivered to the specimen via a gradient refractive index (GRIN) lens. The fluorescence emitted from the imaged specimen returns through the same path to a 535/50 nm bandpass filter and an achromatic doublet lens that focuses the image onto a CMOS sensor of size 640×480 pixels (Fig. 2A-B, [33]). Data acquisition is coordinated by a printed circuit board (PCB) between the microscope and the computer (Fig. 2C, [33]). The miniature microscope can image at a frame rate up to 100 Hz, has a working distance of 150–200 µm, depending on the focal plane, and its lateral resolution is 2.5–2.8 µm.

In order to image a superficial skin blood vessel in a moving animal, we coupled the miniature microscope to a dorsal skinfold window chamber (DSWC) on the back of a mouse. The DSWC is an aluminum chamber that can be implanted surgically in the skin of the back of the mouse and give access to superficial vessels of skin and smooth muscle layer via a protective glass coverslip. [34] Since the miniature microscope was designed for imaging at a working distance of 200 µm, we chose a coverslip harboring a thickness of 55–80 µm. To couple the miniature microscope to the DSWC, we designed a custom u-shaped holder (Fig 2D, Fig. S1) that serves two functions: (1) to position the miniature microscope in the x-y plane of the window chamber on top of a superficial blood vessel of size up to 150 µm diameter, by rotation around the axis of the DSWC main screw, (2) to maintain the miniature microscope in focus, by securing its position along the z-axis (determined using an x-y-z-stage) via the side screw of the holder (Fig. 2D). The miniature microscope weight is less than 2 g, the holder machined in lightweight titanium will weigh less than 1 g, amounting the total weight of the whole mIVM system to less than 3 g.

### Proof of principle imaging of CTCs in a mouse blood vessel

In order to assess the mIVM capabilities to image the 4T1-GL cell line, we first imaged these cells *in culture* using the miniature microscope mounted on an x-y-z stage. We imaged our stably expressing 4T1-GL cell line under three different conditions, in order to maximize the green fluorescent signal-to-background ratio for an optimal detection of every single cell using the mIVM. We first imaged 4T1-GL with or without additional transient transfection with the GFP-Luc2 DNA construct (Fig. 2E). Based on their fluorescence using the miniature microscope, we could clearly distinguish single cells in both cases, but transiently transfected 4T1-GL cells did not appear brighter than stably transfected 4T1-GL cells (Fig. 2E-F). We then labeled 4T1-GL cells with 10 µM of a bright green fluorescent dye, carboxyfluorescein (CFSE), which gave the highest signal-to-background ratio with the miniature microscope when compared to stably transfected and transiently transfected 4T1-GL cells (Fig. 2F), allowing to clearly distinguish every single cell. The dose of dye used is within the dose range recommended by the manufacturer that should not affect cell viability significantly. Based on this observation, we chose to label 4T1-GL cells with CFSE prior to injecting them in animals, in order to maximize their *in vivo* fluorescence signal for mIVM single cell imaging.

**Figure 3:**
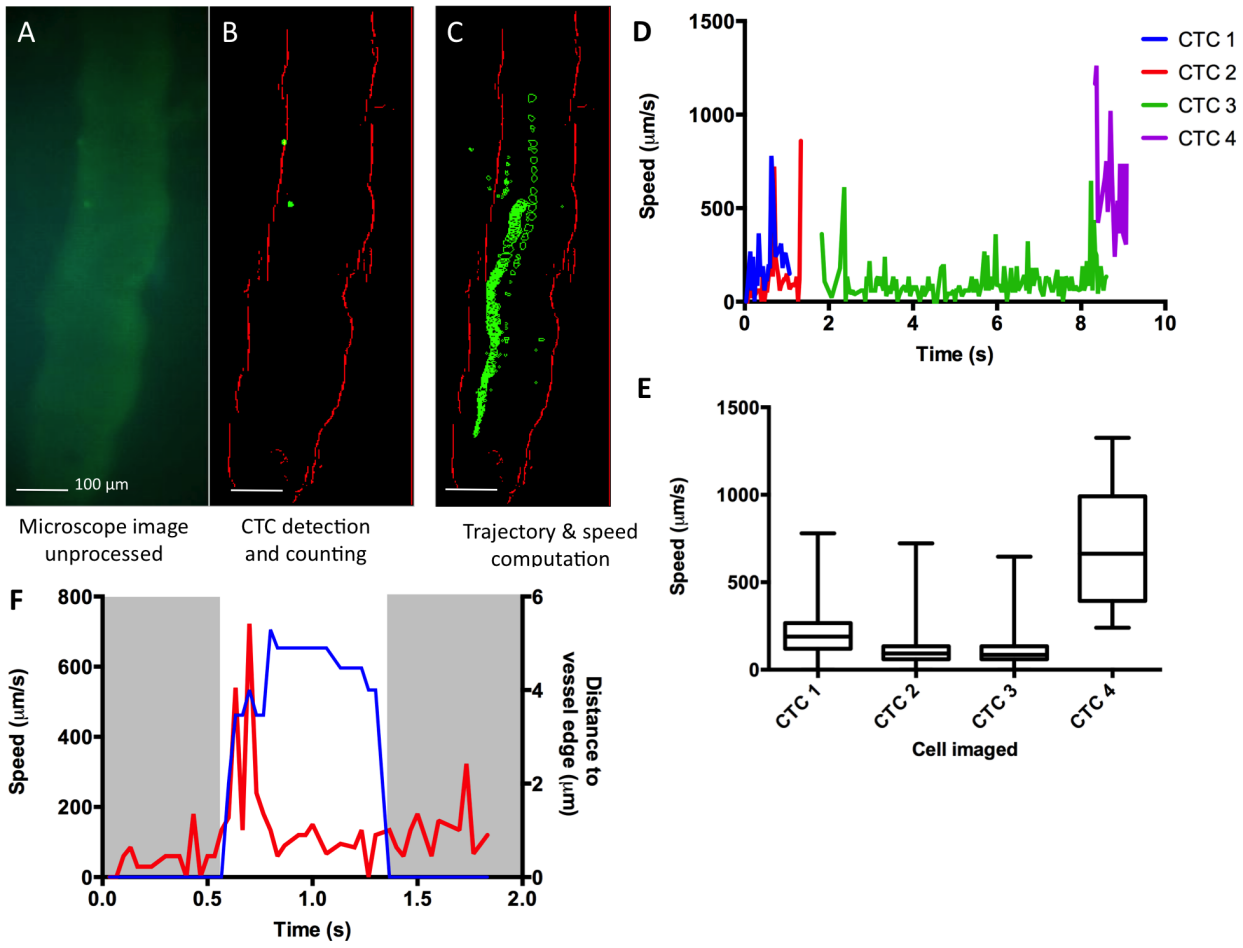
*In vivo* CTCs imaging using miniature mountable intravital microscopy (mIVM) method. (**A**, **B**, **C**) *In vivo* imaging of CTCs using the mIVM after systemic injection of FITC-dextran for vessel labeling followed by injection of 1×10^6^ 4T1-GL labeled with CFSE. (**A**) Raw image from the miniature microscope. (**B**) Image processed by our MATLAB algorithm for detection of CTCs and vessel edges. (**C**) Computing of CTCs trajectories within the blood vessel. (**D**) Quantification of the speeds of CTCs over time as imaged in Movie S1, and (**E**) corresponding average speeds per CTC, plotted as box and whiskers where the box extends from the 25th to 75th percentiles and the whiskers extend from the minimum to the maximum speed values measured. (**F**) For the slowest CTC – CTC2 on (**D**, **E**) – details of the speed of the cell over time (red curve) and the corresponding location of the cell relative to the vessel edge (blue curve).

We first assessed the mIVM performance *in vivo*, by imaging CTCs in a model where a bolus of green fluorescent CTCs was directly introduced in the animal’s bloodstream. To image the mouse’s blood vessels, we intravenously injected low levels of green fluorescent FITC-dextran dye (50 µL at 5 mg/mL). We focused the mIVM system on a 150 µm thick superficial skin blood vessel apparent in the DSWC. Then we tail-vein injected 1×10^6^ CFSE-labeled 4T1-GL cells. In an anesthetized animal, using the mIVM, we were able to observe the circulation of 4T1-GL during the first minutes after injection, as seen on Movie S1 acquired in real-time and shown at a 4x speed. This result confirmed our ability to detect CTCs using the mIVM system. To characterize their dynamics based on the movie data acquired (Movie S1), we developed a MATLAB algorithm to process the mIVM movies, to define vessel edges, identify and count CTCs, as well as compute their trajectory (Fig. 3B-C). This algorithm was used to (1) perform basic operations (background subtraction, thresholding) on the raw data then (2) apply filtering operations to define vessel edges, (3) apply a mask to identify cell-like objects matching the appropriate fluorescence level and size, and (4) count CTCs and map their trajectory (Fig. 3B-C, Movie S1). We also computed the speed of CTCS that were identified by our algorithm (Fig.3D-E). All of the CTCs observed had average speeds <1 mm/s but some CTCs (CTC2, average speed  =  123.6 µm/s, Fig. 3E) had much lower speeds than others (CTC4, average speed  =  704.7 µm/s, Fig. 3E). We also observed that the CTCs moving faster had a trajectory located at the center of the vessel while slower CTCs were closer to the vessel edges. For the slowest CTC, we computed its speed as observed in single frames and related it to its distance to the vessel edge (Fig.3F). We observed that when the CTC was in contact with the vessel edge, its speed would be extremely low (< 200 µm/s), while the speed increased suddenly, up to 722.5 µm/s as the cell detached from the edge of the vessel (t = 0.58s, Fig. 3F). These observations indicate that some CTCs are possibly rolling along the edges of the blood vessels, a mechanism known to facilitate extravasation. [36].

### Continuous dynamics of CTCs over 2 hours in the experimental metastasis model

**Figure 4:**
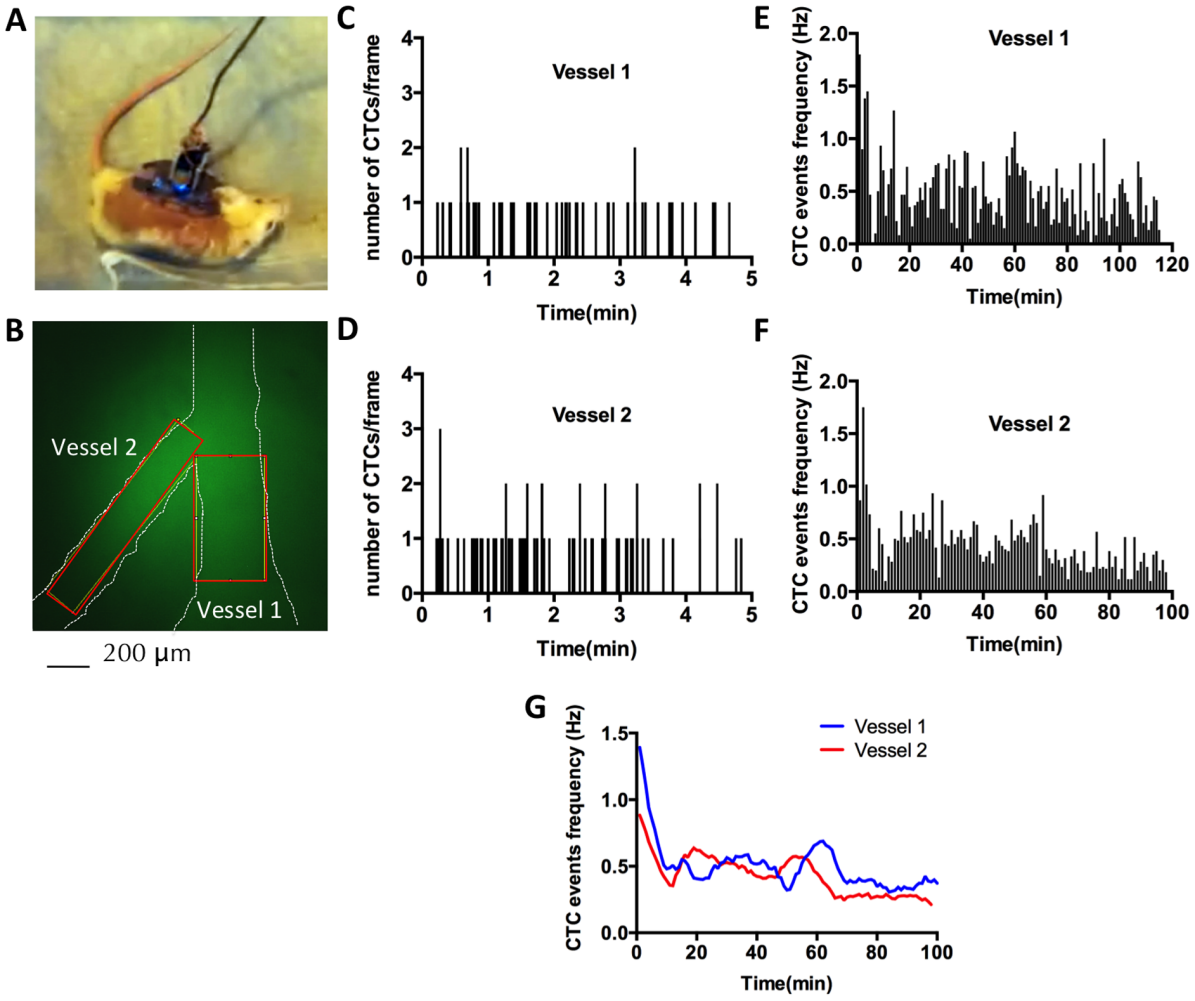
Imaging of circulating tumor cells in an awake, freely behaving animal using the mIVM. (**A**) Photograph of the animal preparation: Following tail-vein injection of FITC-dextran for vessel labeling and subsequent injection of 1×10^6^ 4T1-GL labeled with CFSE, the animal was taken off the anesthesia and allowed to freely behave in its cage while CTCs were imaged in real-time. (**B**) mIVM image of the field of view containing two blood vessel, Vessel 1 of 300 µm diameter and Vessel 2 of 150 µm diameter. (**C**, **D**) Quantification of number of CTCs events during 2h-long awake imaging, using a MATLAB image processing algorithm, in Vessel 1 (**C**) and Vessel 2 (**D**). (**E**, **F**) Computing of CTC dynamics: average CTC frequency (Hz) as computed over non-overlapping 1 min windows for Vessel 1 (**E**) and Vessel 2 (**F**) and (**G**) Second-order smoothing (10 neighbor algorithm) of the data presented in (**E**, **F**)**.**

We next demonstrated imaging of a blood vessel for over 2 hours in an awake animal. A DSWC bearing animal was anesthetized by isofluorane inhalation, and as previously described, received an injection of low levels (50 µL at 5 mg/mL) of plasma-labeling dye FITC-dextran to visualize blood vessels. Subsequently, the mIVM system was focused on an area containing two vessels of 150 and 300 µm diameter (Fig. 4B) and affixed onto the DSWC. After tail-vein injection of 1×10^6^ CFSE-labeled 4T1-GL cells, the animal was allowed to wake up and freely behave in its cage (Fig. 4A, Movie S2), while the mIVM was continuously recording movies of CTCs circulating in both vessels, for 2 hours. Using the MATLAB algorithm described previously, we computed CTC dynamics over time (Fig. 4C-F). As illustrated in Fig. 4C-D, each CTC event (defined as the number of CTCs detected in one movie frame) was recorded in each vessel and the CTC dynamic data over 2 hours was plotted as CTC event frequency for each vessel (Fig. 4E-F). When comparing the smoothed CTC event frequency curves for both vessels, we observed a rapid drop (by 58–65%) of CTC frequencies during the first 10 minutes post-injection, followed by a relatively slow decrease (by 23–48%) of CTC frequency over then next 90 minutes (Fig. 4G). This slow-decrease phase is punctuated by 20–25min long periods of local increases of CTC frequencies, observed as bumps in the decreasing curve. We concluded that the half-life of 4T1-GL CTCs in circulation is 7–9 min post-injection, but that 25% of the CTCs injected are still circulating at 2 hours post-injection. These results demonstrate the feasibility of continuous imaging of CTCs over two hours in an awake, freely behaving animals, using the mIVM system and its capability, together with the MATLAB algorithm, for analyzing CTC dynamics.

## Discussion

In this study, we explored the possibility of using a portable intravital fluorescence microscopy strategy to study the dynamics of circulating tumor cells in living subjects. Using non-invasive bioluminescence and fluorescence imaging, we established an experimental mouse model of metastatic breast cancer and showed that it leads to multiple metastases and the presence of CTCs in blood samples. We utilized a novel miniature intravital microscopy (mIVM) system and demonstrated that it is capable of continuously imaging and computing the dynamics of CTCs in awake, freely behaving mice bearing the experimental model of metastasis.

Besides other advantages described previously, [33] the mIVM system presented here offers three major advantages over conventional benchtop intravital microscopes: (1) it presents a low cost alternative to IVM that is easy to manufacture in high number for high throughput studies (multiple microscopes monitoring multiple animals in parallel), (2) its light weight and portability allow for *in vivo* imaging of blood vessels in freely behaving animals, (3) overcoming the requirement for anesthesia is a novel feature that allows us to perform imaging over extended periods of time, making it ideally suited for real-time monitoring of rare events such as circulating tumor cells. For many applications, mIVM might still be a complementary technique to IVM. However, for CTC imaging, mIVM presents clear advantages when compared to conventional IVM: mIVM is ideally suited for imaging CTCs as it fulfills the needs for (1) cellular resolution, (2) a large field-of-view, (3) a high frame rate and (4) continuous imaging without anesthesia requirements.

The current approach developed here to image CTCs presents several limitations. First of all, due to the current single-channel imaging capabilities of the mIVM, a green fluorescent dye (FITC-dextran) was needed in low concentrations in order to focus the microscope onto blood vessels, but hampered the visualization of eGFP expressing CTCs. Indeed, even though the eGFP expression in the cancer cells was very strong and sustained (Fig. 1B-C), the signal-to-background ratio by mIVM imaging *in vitro* was relatively low (< 2; Fig. 3C). Since the mIVM excitation source is based on a LED, this was expected. However, since a higher signal-to-background ratio was required in order to detect CTCs in the background of FITC-dextran circulating in plasma, we decided to label the cancer cells with a bright green fluorescent dye in addition to reporter gene expression which provided sufficient signal to background to image single 4T1-GL cancer cells both *in vitro* (Fig. 2F) and *in vivo* in the background of FITC-dextran (Fig. S2A). However, even though we were able to image CTCs circulating *in vivo* using the mIVM, there might be a possible signal-to-background issue limiting our capability to image all the CTCs circulating in a vessel.

Labeling the cells exogenously with a fluorescent dye would not be amenable to the study of CTCs in an orthotopic mouse model of metastasis, where CTCs would spontaneously arise from the primary tumor. In order to avoid this issue, we envision two solutions. The first one, based on our current imaging setup requires waiting for 1–2 hours post - FITC-dextran injection to start imaging CTCs. Indeed we have observed that the FITC-dextran is almost completely cleared of blood vessels 2h-post injection (Fig. S2B). The second approach rely on the next-generation design of mIVM setups capable of multicolor imaging, similarly to benchtop IVM systems. Using a dual-channel mIVM currently under development, the blood plasma could be labeled using a dye with different excitation/emission spectrums and circumvent the need for double labeling of the CTCs.

Another limitation of the mIVM is its penetration depth/working distance of max. 200 µm, [33] allowing imaging through a 55–80 µm thick coverslip of superficial blood vessels of diameter up to 145 µm (the skin layer was removed as part of the window chamber surgery). For the 150 µm and smaller vessels – which are typical vessel sizes for IVM setups – our miniature microscope is capable of imaging the entire blood vessel’s depth. However in the case of the largest vessel of 300 µm diameter imaged here (Fig. 4B), the penetration depth might have limited our capabilities to image all the CTCs circulating in this vessel. Therefore, the mIVM system is not intended to measure deep vessels, and should focus on smaller superficial blood vessels.

In this manuscript, we do not intend to image all the CTCs circulating in a mouse’s bloodstream, nor do we intend to image all the CTCs circulating in a particular vessel, as there might be depth penetration, fluorescence variability and signal-to background issues preventing us from recording all the CTCs events. Instead, we demonstrate here that we can image a fraction of the CTCs circulating in a particular superficial blood vessel. Assuming that the blood of the animal is well-mixed, the circulation dynamics of this fraction are representative of the circulation dynamics of CTCs in the entire blood pool. This assumption is common to all existing CTC detection methods that detect CTCs in a fraction of the entire blood pool (a blood sample, or an imaging time-window for in vivo flow cytometers) and/or detect a fraction of all the *bona fide* CTCs that are expressing a specific marker (e.g. EpCAM, CK, melanin, a fluorescent label). Since we are focusing on one small superficial blood vessel, we are not able to detect all the CTCs injected but only a small fraction of them, whose circulation dynamics we believe to be reflective of the dynamics of all the CTCs in this mouse model. In order to estimate this fraction and therebye estimate the sensitivity of our method, we estimated the total number of CTCs events detected over 2 hours: over 2 hours, we were able to detect an average of 2930 CTC events in a vessel, out of 1×10^6^ cells injected, that is 0.29% of the CTCs injected. However, we believe that this number is not able to really reflect the true sensitivity of our method since the number of CTC events detected is dependent on (1) the size of the blood vessel imaged, (2) the relative location of the blood vessel in the circulation system, (3) the unknown fraction of CTCs circulating multiple times, that are therefore counted multiple times, (4) the unknown fraction of CTCs dying, (5) the unknown fraction of CTCs arresting/extravasating in organs. All these parameters require a complex mathematical model to relate the number of CTCs detected over a period of time to the actual sensitivity of our method at detecting CTCs.

As far as the specificity of our method is concerned, we are assuming here that only the cancer cells labeled with CFSE will generate a strong green fluorescence signal. We acknowledge that there could be some autofluorescence issues that would make tissue appear fluorescent as well. Therefore, we programmed our CTC detection algorithm to only count as a cell an object of the right fluorescence level harboring a circular shape of the right diameter (10–20 µm). Furthermore, any fluorescent object that is not moving at all over the imaging window (10 min – 2h) is going to be considered as background. We tested and optimized the algorithm on small imaging datasets before applying it to a larger dataset as presented on Fig.4.

This study provides a proof-of-principle for mIVM imaging of CTCs in awake animals. However, we only explored the experimental model of metastasis, where 4T1 metastatic cancer cells are injected into the tail vein and allowed to circulate and seed metastasis sites. In this model, we imaged CTCs as they circulate during the first 2 hours post-injection. We were able to identify key features of the dynamics of CTCs: variations in speed and trajectory, rolling phenomenon when CTCs are in contact with the vessel edges (Fig. 3), half-life of CTCs in circulation in awake animals, representative fraction of CTCs still circulating 2 hours post-injection in awake animals (Fig. 4). Our measurements of the half-life of 4T1-GL cells (7-9 min) is in the same range than previous half-life measurements done on other metastatic cancer cell lines as measured with IVM methods. [23], [37] Similarly the rolling phenomenon we observed with the 4T1-GL cells has been demonstrated and studied in-depth in previous litterature. [36] We were not able to image CTCs in the same mice around day 12, where the re-circulation of CTCs seems to happen because at that time, animals were showing signs of distress and needed to be sacrificed. It would be interesting to apply the mIVM method to a breast cancer model where the primary tumor is naturally shedding CTCs into the circulation. We envision that the mIVM will be particularly useful to explore the dynamics of CTCs in orthotopic metastasis models, since it has the ability to continuously monitor a blood vessel for sporadic and relatively rare CTC shedding events.

Our current mIVM setup is weighing less than 3 g, and is mounted on a titanium DSWC weighing less than 3 g, amounting the total weight to less than 20% of the mouse’s body weight (for a 30 g mouse). This setup would certainly be considered heavy for long term imaging of the superficial skin and smooth muscle on the back of the mouse. For longer imaging sessions, we envision that the setup would be placed on a cranial window chamber instead of the DSWC. Our collaborators, Ghosh et al., have previously demonstrated the feasibility of brain imaging using the mIVM in a cranial preparation. [33] This preparation could be used similarly to image CTCs in the brain and alleviate the weight of the setup on the skin. Another strategy to offset the weight of the system is to use a counterweight system in the cage, similarly to the one used for the RatCAP head-mounted PET imaging system. [38]

We describe here how mIVM imaging allows enumerating CTCs as they circulate in a mouse’s bloodstream. This *in vivo* CTC enumeration method offers several advantages over *in vitro* interrogation of CTCs in blood samples. First of all, as the imaging is relying on the endogenous expression of the eGFP protein by the CTCs, there need not be reliance on a given CTC marker for CTC imaging or capture. Furthermore, the blood volume that can be analyzed by continuously imaging a blood vessel can potentially be much larger than that of a blood sample, enabling the potential capture of even more rare events. Assuming a blood flow speed of 1 mm/sec in a blood vessel of 100 µm diameter (typical parameters measured in our mIVM experiments), we estimated that we are able to analyze 28 µL of blood per hour. If we perform continuous imaging over 24 hours, we will be able to sample 672 µL of blood. Over 1 week, we will be able to sample over twice of the total mouse blood volume (∼2 mL), versus 5% as allowed per veterinary guidelines for blood sampling. If we image larger vessels with higher frame rates, we will be able to achieve even higher blood volume analysis. The current mIVM system will also be particularly useful to image tumor cells as they are leaving a primary tumor and entering the bloodstream – this can be achieved by implanting a primary tumor at the site of the dorsal skinfold window chamber. This strategy will also increase the probability of observing naturally occurring CTCs.

Previously, other *in vivo* CTC imaging methods have been used to interrogate CTCs in living animals, namely *in vivo* flowmetry [23] and multiphoton intravital flow cytometry [24]. Both techniques are benchtop systems and have been able to detect single CTCs as they are flowing in a mouse’s ear blood vessels. Multiphoton microscopy harbors much higher signal-to-background ratios (∼22) than mIVM for detecting dye-labeled cells (∼2). [24] However, since both methods are based on time-consuming laser-scanning, they had to rely on a one-dimensional line scanning through a slit in a blood vessels in order to detect fast flowing CTCs. Our mIVM method has the advantage of combining high speed detection (up to 100 Hz) and two-dimensional imaging. In our mIVM setup, an image of the detected CTCs can be formed, to confirm that the signal detected is indeed coming from CTCs. Moreover, thanks to its miniaturization, our mIVM system is the first setup we know of allowing to image CTCs in awake, freely-behaving animals. Eventual use of these and related devices to monitor CTCs in humans (e.g., for monitoring for tumor recurrence) may also be possible by combining these devices with implantable patches that periodically inject fluorophores that target CTCs for continuous monitoring strategies.

To shed light on the potential clinical relevance of CTCs, complex questions about tumor metastasis need to be answered: (1) how and when a breast tumor infiltrates the bloodstream, (2) how inefficient the process of metastasis is for a particular carcinoma and (3) which properties of CTCs enable them to successfully colonize distant organs. Here we have demonstrated that our new mIVM system is capable of continuously imaging blood vessels for CTCs in awake animals. Our system has the potential to shed light on some of the fundamental questions raised above. We are currently exploring the possibility of using an optoelectronic commutator for long term use of the mIVM system in awake freely moving subjects as well as developing a real-time analysis algorithm that will only keep and store the data corresponding to CTCs events. This method will enable the *in vivo* long term study of CTCs dynamics in orthotopic mouse models of metastasis.

## Conclusions

We have demonstrated here how a new technology, miniature intravital microscopy, can be applied to the study of metastatic circulating tumor cells dynamics in living awake animals. We expect that miniature intravital microscopy will become a useful technique for the precise characterization of the long-term dynamics of CTCs in vivo. New developments in miniaturization of the system will undoubtedly improve the performance of the technique. The introduction of dual fluorescence channels will provide better signal-to-noise ratio by allowing to image blood plasma and CTCs on separate imaging channels. The use of lighter materials like titanium and of counterbalance arm systems will permit to design lighter systems that an animal could wear continuously for several days.

## Supporting Information

Figure S1 **U-shaped holder. (A)** Pictures of the elements of the mIVM system: U-shaped holder and miniature microscope. **(B)** Schematic of the U-shaped holder and its function. The microscope securing screw helps to secure the miniature microscope in the holder. The window chamber securing screw secures the holder onto the window chamber. Scale bars, 5 mm (**A**,**B**).
(TIF)

Figure S2 **Signal-to-background measurements.** (**A**) Quantification of fluorescence intensity of CTCs and background as measured on Movie S1. Average fluorescence intensity was measured over 12-164 frames for CTCs and over 29 frames for the background intensity of the blood vessel (named “B”). (**B**) Example of mIVM images obtained with the mIVM immediately following injection of 50 µL at 5 mg/mL of FITC-dextran as well as 2 hours following injection. The images show the extravasation of the dye resulting in lower background signal in the vessel after 2 hours imaging.
(TIF)

Movie S1 **Raw Movie from mIVM showing mIVM imaging of CTCs circulating following i.v. injection of the cells (left panel).** The movie was acquired in real-time and is shown at a 4x speed. Corresponding MATLAB image processing using in-house algorithm (right panel).
(MP4)

Movie S2 **Video of an awake BALB/C mouse bearing the miniature microscopy setup and freely behaving in its cage, while the microscope was recording data.**
(MP4)
