# Supplementary figures and images for: Imaging Circulating Tumor Cells in Freely Moving Awake Small Animals Using a Miniaturized Intravital Microscope
Source: PLoS One. 2014 Jan 31;9(1):e86759. doi: 10.1371/journal.pone.0086759 (PMC3908955; doi:10.1371/journal.pone.0086759)

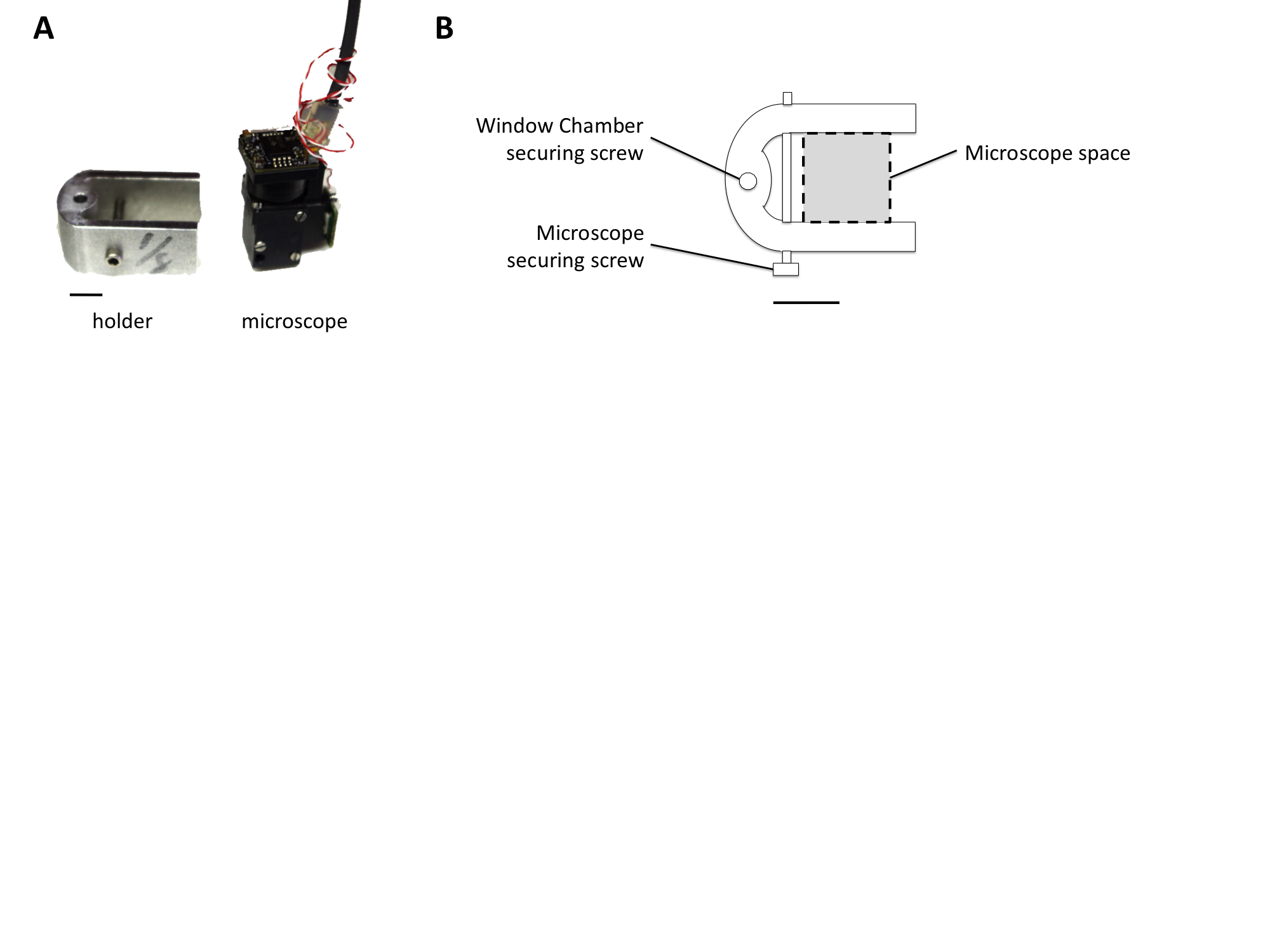

Supplement: Figure S1 — U-shaped holder. (A) Pictures of the elements of the mIVM system: U-shaped holder and miniature microscope. (B) Schematic of the U-shaped holder and its function. The microscope securing screw helps to secure the miniature microscope in the holder. The window chamber securing screw secures the holder onto the window chamber. Scale bars, 5 mm (A,B). (TIF) [file pone.0086759.s001.tif]

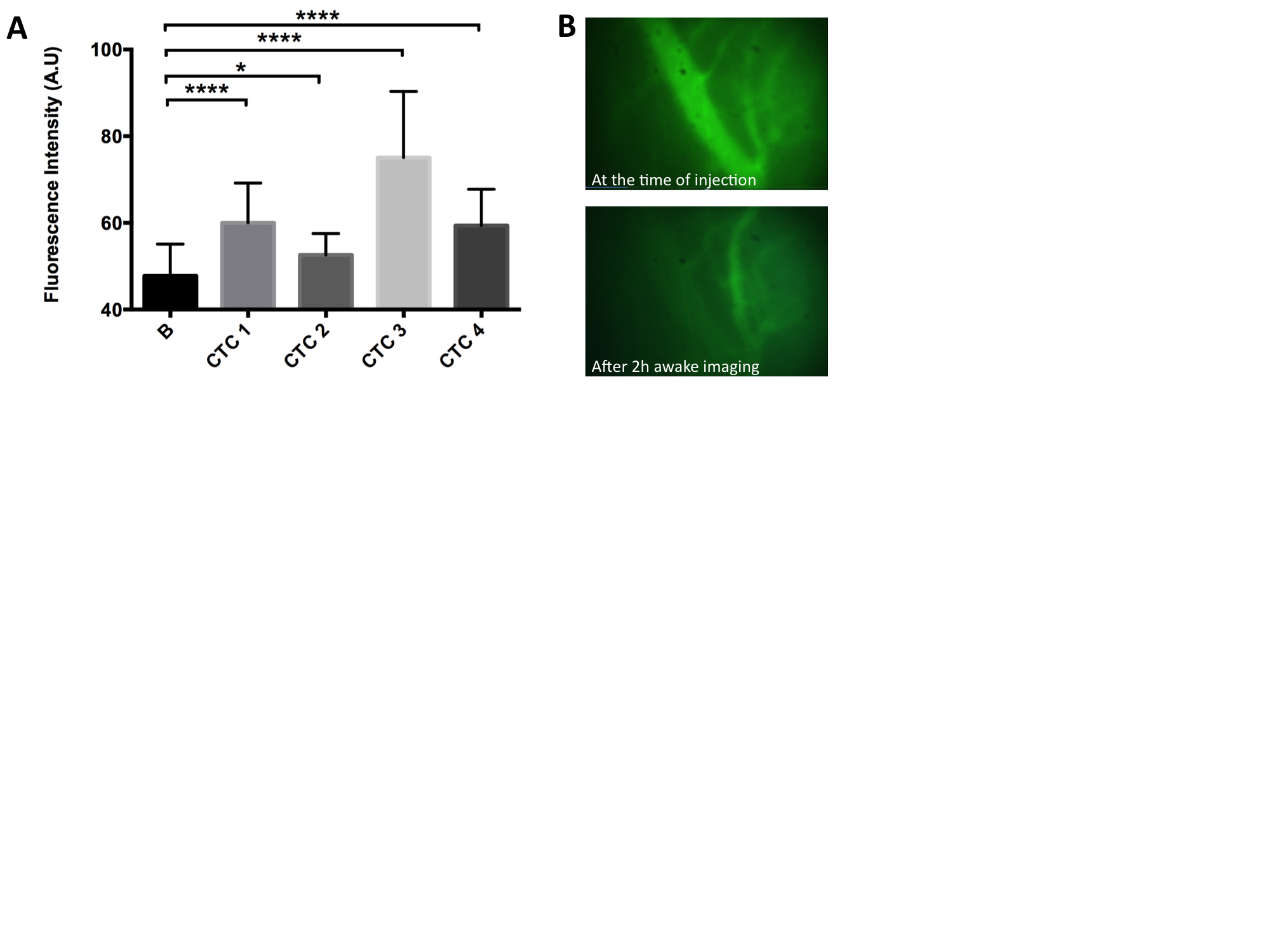

Supplement: Figure S2 — Signal-to-background measurements. (A) Quantification of fluorescence intensity of CTCs and background as measured on Movie S1. Average fluorescence intensity was measured over 12-164 frames for CTCs and over 29 frames for the background intensity of the blood vessel (named “B”). (B) Example of mIVM images obtained with the mIVM immediately following injection of 50 µL at 5 mg/mL of FITC-dextran as well as 2 hours following injection. The images show the extravasation of the dye resulting in lower background signal in the vessel after 2 hours imaging. (TIF) [file pone.0086759.s002.tif]
